# Multi-Level Factors Associated with Social Participation among Stroke Survivors: China’s Health and Retirement Longitudinal Study (2011–2015)

**DOI:** 10.3390/ijerph16245121

**Published:** 2019-12-15

**Authors:** Yi Cai, Samuel D. Towne, C. Scott Bickel

**Affiliations:** 1 Department of Global Health, Wuhan University School of Health Sciences, 115 Donghu Road, Wuhan 430071, China; 2 Wuhan University Global Health Institute, 8 Donghu south Road, Wuhan 430072, China; 3 Department of Health Management & Informatics, University of Central Florida, 4000 Central Florida Blvd, Orlando, FL 32816, USA; Samuel.Towne@ucf.edu; 4 Disability, Aging, and Technology Cluster, University of Central Florida, Orlando, FL 32816, USA; 5 Department of Environmental and Occupational Health, School of Public Health, Texas A&M University, College Station, TX 77843, USA; 6 Southwest Rural Health Research Center, Texas A&M University, College Station, TX 77843, USA; 7 Center for Population Health and Aging, Texas A&M University, College Station, TX 77843, USA; 8 Department of Physical Therapy, Samford University School of Health Professions, CHS Building 2 2159, 800 Lakeshore Drive, Birmingham, AL 35229, USA; cbickel@samford.edu

**Keywords:** social participation, post-stroke, community building

## Abstract

Background: This study aims to examine the impact of individual-level and community-based factors on popular social participation activities of Chinese middle-aged and older adults post-stroke. Methods: Sub-samples of survivors of stroke (2011: n = 413, 2013: n = 395, 2015: n = 441) recruited by the China Health and Retirement Longitudinal Study (CHARLS) were included in the analysis. Zero-inflated Poisson and multi-level logistic regression models were used to explore factors associated with social participation. Results: More than half of individuals (55.0%) had no social participation and 23.4% participated in multiple social activities. The most popular social activities that individuals participated in were interacting with friends (32.6%) and going to a community club to play table games (22.7%). Multiple individual-level factors were negatively related to social participation (e.g., depressive symptoms and multiple measures of functional limitations) while the allocation of an outdoor exercise facility in the community/village was positively associated with going to a community club to play table games. Conclusion: Stroke survivors are at high risk of limited social participation. Policymakers and other key stakeholders should consider community design among other potential solutions when identifying ways to link at-risk stroke survivors to both opportunities for rehabilitation (e.g., physical function) and social participation.

## 1. Introduction

Stroke is a leading cause of death and disability among middle-aged and older adults throughout the world. In China, the adjusted stroke prevalence was 2.06% in adults aged 40 years and older with an overall annual increase of 8.3% [1]. A Chinese national survey recently reported the long-term disability rate in people five years after stroke was 45% [2]. Thus, these survivors of stroke likely have significant restrictions in social participation that may be associated with lower quality of life and a higher burden of caregiving. In addition, recent evidence suggests a link with social isolation and an increased risk of stroke [3], which makes this an important factor to consider.

Social participation is considered a key component for returning to function for individuals post-stroke in the International Classification of Functioning, Disability, and Health (ICF) model [4]. A meta-ethnographic review indicated that it is important for survivors of stroke to engage in meaningful self-selected social activities [5]. Studies have found that the preferred or popular social activities were different among survivors of stroke based on the country in which they are living [6,7,8]. Additionally, there is no consensus on a definition of social participation [9,10].

Social participation among older adults is associated with one’s culture and community. Researchers are increasingly interested in identifying popular social activities that survivors of stroke engage in across diverse cultures [6,7]. For example, in China, it is a cultural tradition that older Chinese parents reside with adult children and provide assistance to family members, such as taking care of grandchildren [11]. Community-dwelling older Chinese individuals have reported preferred activities such as square dancing and table gambling games such as mahjong, which is played in groups of four [12,13]. However, there is insufficient research examining social participation and the impact of individual-level and community-based factors among survivors of stroke in China.

Previous studies have reported that social participation was significantly associated with individual-level factors such as socio-demographic factors [5], having comorbidities [14], physical function [15], being depressed [9], and having restrictions with participation in activities [16]. However, these studies investigated survivors of stroke at different post-stroke phases from multiple settings, were not based on nationally representative longitudinal samples, and often report inconsistent findings, with minimal inclusion of community-based factors that also play a role in each stage of the recovery process post-stroke.

Thus, the aim of this study is to examine the impact of individual-level and community-based variables on popular social participation among Chinese middle-aged and older community-dwelling adults that were stroke survivors. The conceptual framework is linked to the ICF, which was endorsed by the World Health Assembly in 2001 to measure health and disability at both individual and population levels. In the current study, we used data collected by the China Health and Retirement Longitudinal Study (CHARLS) between 2011 and 2015 [17] by not only examining factors related to the domains of bodily function and personal factors but also the domain of environmental factors.

## 2. Methods

### 2.1. Data and Study Population

This study used three waves of data from the CHARLS [17]. The CHARLS is a nationally representative longitudinal survey of the middle-aged and older adult (inclusive of ages 45 years old and over) in China, along with their spouses, which includes individual-level, household-level, and community-level variables related to health and retirement. In the CHARLS cohort, if the chosen person was 45 years of age or older, the research group interviewed both that person and his or her spouse. In the first wave of 2011, the final sample was 17,708 individual participants, including 10,069 main respondents randomly selected after the randomization procedures for the survey and 7639 spouses of the main respondents. The national survey has been conducted every two years since 2011. Follow-ups not only track baseline respondents but also non-response information. A newly recruited sample (2013 and 2015) was added to replenish the nationally representative sample.

The baseline survey was conducted in 2011 on 17,708 participants, of which the sample of participants who were stoke survivors was 413, accounting for 2.33% of all participants. The follow-up surveys in 2013 and 2015 recruited 18,605 and 21,095 participants, respectively. The sample of stroke survivors in 2013 was 395 (2.12%), of which the follow-up sample was 335 and the newly recruited sample included 60 stroke survivors. The sample in 2015 was 441 (2.09%), of which the follow-up was 430 and the newly recruited sample contained 11 stroke survivors.

### 2.2. Measures

#### 2.2.1. Dependent Variables

##### Social Participation

The CHARLS asked participants to choose which social activities they participated in during the past month, including: (1) “Interacted with friends,” (2) “Played mahjong, chess, cards, or went to a community club to play table games,” (3) “Provided help to family, friends, or neighbors who do not live with you and who did not pay you for the help,” (4) “Went to a sport, social, or other kind of club,” (5) “Took part in a community-related organization,” (6) “Done voluntary or charity work,” (7) “Cared for a sick or disabled adult who does not live with you and who did not pay you for the help,” (8) “Attended an educational or training course,” (9) “Stock investment,” (10) “Used the Internet,” and (11) “others if none of above.” We generated dependent variables of social participation as a dichotomous variable (participating in at least one type of these activities in the past month = “1” versus no participation = “0”) and a continuous variable (the number of social activities one participated in).

#### 2.2.2. Independent Variables

##### Personal Factors

Personal factors included socio-demographic variables, including: age, gender, marital status (married and living with spouse = “1”; others = “0”), education level (“no formal education,” “did not finish primary school,” “sishu (home school),” and “primary school” = “1”, others = “0”), and residency (rural residency = “1”, urban residency = “0”). Age and gender were included in adjusted analyses.

### 2.3. Health Status

In order to measure general health including physical and mental health and disability, we used the following variables: self-rated health, comorbidity (one or more chronic conditions), having depressive symptoms, and disability of Activities of Daily Living (ADLs)/Instrumental Activities of Daily Living (IADLs). We recoded original responses for each of the items and generated new variables for statistical analysis. One’s self-rated health was coded as a dichotomous variable (fair and good = “1”, poor = “0”). Comorbidity was coded as a continuous variable with depressive symptoms, measured by the 10-item Center for Epidemiologic Studies Depression Scale (CESD-10), which was coded as a dichotomous variable (a score of 10 and higher = depressive symptoms “1”, a score of lower than 10 = no depression “0”) [18]. The ADLs/IADLs disability were coded as dichotomous variables (needing help with at least one item of ADLs/IADLs disability coded as disabled = “1”, versus none (no disability) = “0”).

### 2.4. Physical Activities

Physical activities were measured by self-reported physical functions. The participants were asked to report different types of physical functioning, including: (1) “Do you have any difficulty with running/jogging about 1 km?”, (2) “Do you have any difficulty with walking 1 km?”, (3) “Do you have any difficulty with walking 100 m?”, (4) “Do you have any difficulty with getting up from a chair after sitting for a long period?”, (5) “Do you have any difficulty with climbing several flights of stairs without resting?”, (6) “Do you have any difficulty with stooping, kneeling, or crouching?”, (7) “Do you have any difficulty with reaching or extending your arms above shoulder level?”, (8) “Do you have any difficulty with lifting or carrying weights over 5 kg?”, and (9) “Do you have any difficulty with picking up a small coin from a table?”. Each physical function was coded as a dichotomous variable (“no, I don’t have any difficulty” = “1”, “I have difficulty but can still do it,” “yes, I have difficulty and need help,” or “I cannot do it” = “0”).

### 2.5. Community-Based Factors

In addition to the variable measuring rurality (rural or urban), the CHARLS asked about other community-based characteristics including asking one person who is familiar with the community to answer questions in the community questionnaire. These persons are government staff who are in charge of the administration of communities or villages. The Chinese government sets up a residential committee in each residential urban community or rural village and assigns one governmental staff in charge of the management. They are the most fundamental levels of the Chinese government’s administration system.

There was a question about the allocation of community facilities, such as outdoor exercise facilities, game rooms, dancing or other exercise teams, activity centers for the elderly, nursing homes, and others. On 15 February 2011, the State Council of the Chinese government issued the National Fitness Program (2011–2015) that allocated fitness facilities in at least 50% of urban communities and rural villages. This plan aims to increase space for exercise and remove barriers to accessing public physical environments. Given this health-related public policy implementation, we selected the allocation of outdoor exercise facilities as a proxy for the environmental design by the government to assess its role in social participation, if any.

### 2.6. Statistical Analyses

Data analyses were performed using Stata 14.0 (StataCorp LLC, College Station, TX, USA). Descriptive analyses were used to present sample characteristics and the social activities one participated in, if any. The longitudinal data used in this study allow for assessing changes over time. In order to examine the consistency of the baseline and follow-up samples and compare the changes of the samples’ health-related and functional status over time, ANOVA and Chi-square tests were used to examine differences in social participation and the four sets of independent variables (socio-demographic factors, health status, physical impairments, and allocation of outdoor exercise facilities in the community/village) between 2011 and 2015. Logistic regression, zero-inflated Poisson regression, and multilevel logistic regressions were used to explore impacts on social participation. Because there was a high percentage of study participants with no social participation (more than 50%), the zero-inflated Poisson regression model was used to explore the impact on the number of social activities one participated in. A multilevel logistic regression model was utilized to examine the impact on the probability of social participation at both individual and community levels. An individual weight with a household non-response adjustment and individual non-response adjustment in the CHARLS data was applied in all descriptions and the zero-inflated Poisson regression model. Two means calculated with unweighted data and weighted data have been reported. At each step of multi-level modeling, we estimated goodness of fit indices (deviance statistic, Akaike Information Criterion (AIC), and Bayesian Information Criterion (BIC)) to compare fit across the non-nested models and report the model with the best goodness of fit.

## 3. Results

### 3.1. Sample Characteristics

Demographics of the study participants are presented in Table 1. Most were male (53.3%), and married (71.2%), with a primary school education or lower (70.1%), and living in rural areas (58.2%). The sample was living with 2.5 types of chronic diseases in addition to being a stroke survivor. Most of the sample reported poor health (67.6%) and had disabilities of ADLs (88.5%) and IADLs (79.8%). More than half (68.7%) were rated as having depressive symptoms. Having any difficulty running/jogging about 1 km (79.5%) was the most common physical disability, while having any difficulty picking up a small coin from a table (21.1%) was the least common physical disability. More than half of participants (62.1%) had no social participation. Over time, participants were less likely to rate depressive symptoms ($\chi^{2}$ = 16.48, *p* < 0.001), reported more need of assistance with IADLs ($\chi^{2}$ = 14.94, *p* = 0.001), reported having difficulty walking 100 m more often ($\chi^{2}$ = 21.40, *p* < 0.001), and reported more social participation activities (*F* = 4.24, *p* = 0.015) over time.

### 3.2. Participation of Each Social Activity

Table 2 presents the proportions of participants with different types of social participation between 2011 and 2015. Interacting with friends (range: 22.1% to 32.6%) and going to a community club to play table games (range: 12.6% to 22.7%) were the most frequently reported social activities.

### 3.3. The Number of Social Activities Individuals with Health Issues Participated in

On average, participants having difficulty with climbing several flights of stairs without resting reported the largest number of social activity participated in (2011: mean = 1.01), and those having depressive symptoms reported the smallest number of social activities participated in (2011: mean = 0.46) (Table 3).

### 3.4. The Impact on the Number of Social Activities Individuals Participated in

Study participants who need assistance with ADLs (Coef. = 1.78, *p* = 0.001) were expected to participate in nearly two more types of social activities, and those who need assistance with IADLs (Coef. = 0.98, *p* = 0.001) were expected to participate in nearly one more type of social activity (Table 4). Participants who have difficulty with running or jogging about 1 km (Coef. = 0.46, *p* = 0.005), difficulty with climbing several flights of stairs without resting (Coef. = 0.29, *p* = 0.045), difficulty with reaching or extending your arms above shoulder level (Coef. = 0.32, *p* = 0.010), and difficulty with lifting/carrying weights over 5 kg (Coef. = 0.29, *p* = 0.010) were more likely to report a smaller number of social activities. Those with depressive symptoms were more likely to report a smaller number of social activities that they participated in (Coef. = −0.35, *p* = 0.019).

### 3.5. The Impact on the Probability of Social Participation

Table 5 presents results for the probability of any social participation. There was a higher probability of participating in social activities among participants who needed assistance with ADLs (OR = 5.53, CI: 1.84, 16.56) and IADLs (OR = 2.95, CI: 1.52, 5.72), and lower probability among those who were rated as having depressive symptoms (OR = 0.50, CI: 0.37, 0.67), and having difficulty with walking 1 km (OR = 0.63, CI: 0.46, 0.86) and difficulty with lifting/carrying weights over 5 kg (OR = 0.50, CI: 0.35, 0.69).

In addition, there are other influencing factors on the probability of interacting with friends, such as gender (OR = 0.62, CI: 0.46, 0.83), difficulty with walking 100 m (OR = 1.49, CI: 1.05, 2.08) and difficulty with lifting/carrying weights over 5 kg (OR = 0.58, CI: 0.41, 0.82).

Younger participants (OR = 0.97, CI: 0.95, 0.99) with higher education (OR = 0.53, CI: 0.33, 0.86) were more likely to going to a community club to play table games. However, those with depressive symptoms (OR = 0.36, CI: 0.23, 0.57), and with difficulty reaching/extending arms above their shoulder level (OR = 0.30, CI: 0.17, 0.55), difficulty with lifting/carrying weights over 5 kg (OR = 0.54, CI: 0.32, 0.91), and difficulty with picking up a small coin from a table (OR = 0.50, CI: 0.28, 0.81) were less likely to go to a community club to play table games. The allocation of an outdoor exercise facility in the community/village had positive impacts on the likelihood of participants going to a community club to play table games (OR = 1.51, CI: 1.17, 1.96).

## 4. Discussion

The purpose of this study was to identify popular social participation activities among Chinese middle-aged and older adults post-stroke and explore individual-based and community-based factors that impact social participation using nationally representative longitudinal data. Furthermore, 60% of the sample reported no social participation. In the nearly 40% that did report participation, we identified two types of social activities that Chinese survivors of stroke participated in the most. Females were more likely to interact with friends than males, and older individuals with lower education levels were more likely to go to a community club to play table games than younger individuals with higher education levels. Needing assistances with ADLs and IADLs were facilitators to social participation but having depressive symptoms was a barrier. In addition, having physical impairments of the upper limbs was also a barrier to go to a community club to play table games. The allocation of outdoor exercise facilities in the community/village, which served as a proxy of the community design, had a positive impact on going to a community club to play table games. As such, community design, namely the existence of an outdoor exercise facility, is one aspect of community design that should be further considered by local and national policy makers, given its potential to improve the likelihood of social interaction among potentially vulnerable adults throughout China.

We analyzed the type of social activities that stroke survivors participated in and found that interacting with friends was the most popular social activity. This is likely due to the fact that it is among the more feasible social activities. For example, interacting with friends can include phone or online communication, including the availability of friends to visit even if they are home-bound. The second most popular social activity among the participants was going to a community club to play table games. Previous research has also demonstrated that Chinese older adults were more likely to visit community centers than any other social or cultural venue [19].

The current study is inconsistent with previous studies finding that needing assistance with ADLs/IADLs was associated with a participation restriction [20]. The current study showed evidence that stroke survivors needing assistance with ADLs/IADLs not only had a higher probability of participating in social activities but also participated in more types of social activities. Interacting with friends, which was the most popular activity identified in our study, may be particularly critical to those that are in need of assistance with ADLs/IADLs.

Findings on the impact of physical impairments on social participation post-stroke shed greater light on stroke survivorship among Chinese middle-aged and older adults. This study identified that social participation is associated with physical impairments, which represents upper limb and lower limb functions as well as fine motor function important for walking and other forms of physical activity. Kim et al. designed a community walking training program within the community environment targeting improvements with walking function and social participation among survivors of stroke in South Korea [10]. Their findings indicated the intervention group had greater improvement in walking function and social participation than those in the control group. This is relevant with our findings that demonstrate difficulty with walking at least 1 km but not 100 m was associated with limitations in social participation, which indicated many can at least walk short distances out of the home within the community. Thus, Chinese rehabilitation professionals can use these findings to focus rehabilitation activities on walking short distances within and outside the residence of a person with stroke. Furthermore, smart community design in areas with a higher population density of older adults can ensure neighborhoods are designed to be more age-friendly (e.g., walkable, mixed-use design including multiple utilitarian destinations within short distances of residential buildings). Previous evidence outside of China suggests that older adults living in areas with mixed land use and areas that were more compact had higher levels of walking and that the area within 400 m of their residence is important when considering walking in ones’ neighborhood [21]. This supports the concept of designing more walkable, mixed land use, and overall age-friendly environments.

Findings showed that difficulties with a group of lower-limb functions were generally barriers to interacting with friends but difficulties with a group of upper limb functions were barriers to going to a community club to play table games. These findings imply that stroke survivors might prefer to interact with friends by going out and meeting friends face-to-face. Thus, when they had difficulties with lower-limb functions and could not walk for a certain distance, they might fail to go out and meet up. Stroke survivors with a hobby of playing table games might have a strong motivation to find a way to go out and play games. However, if they had difficulties with upper limb functions especially difficulty with fine motor functioning, they may be unable to play table games especially Mahjong, which is the more popular table game [12]. Further studies are necessary to explore stroke survivors’ preferences in social participation.

There were two factors associated with four dependent variables examined in our study: a proxy of physical function (difficulty with lifting/carrying weights over 5 kg) and a proxy of mental health (having depressive symptoms). Epidemiological studies have showed that grip strength is a predictor of disability in later life [22]. The function of lifting/carrying weights over 5 kg may be a proxy of the grip strength and can predict disability in later life. Thus, when stroke survivors had difficulty with lifting/carrying weights over 5 kg, they may have been more generally disabled and potentially limited in their ability to participate in social activities. Depressive symptoms are widely associated with physical health, mental health, and social factors [23,24,25]. Our study also found that having depressive symptoms was not only negatively associated with the number of social activities one participated in but also the probability of social participation. Thus, intervening in terms of ameliorating depressive symptoms and promoting upper limb’s muscle strength are priorities.

Individual-level and community-based factors had different impacts on two types of popular social participation. Gender had a significant impact on the probability of interacting with friends but not on the probability of going to a community club to play table games. Women were more likely to seek help from friends, which possibly makes them more active in building and maintaining individual relationships, and having a higher social expectation by communicating with significant others instead of men [26]. Thus, a gender difference may be a significant factor that impacts interacting with friends among survivors of stroke.

The importance of environmental factors was also supported in findings indicating individuals living in communities with an outdoor exercise facility had a higher probability of social participation. Designing neighborhoods that include exercise facilities not only provide opportunities for physical activity, but also provide opportunities for social interaction (e.g., with neighbors). One qualitative study showed that intention, ability, and having opportunities for physical activity were determinants of outdoor walking activity [27]. Following the implementation of China’s National Fitness program (2011–2015) [28], the State Council of China initiated a program that aims to allocate fitness facilities in the county/town/village networks in rural areas and within a 15-min walking distance in urban communities between 2016 and 2020 [29]. Thus, it is possible that current gaps in access to outdoor exercise facilities in some residential communities may be lessened in the near future. Continued monitoring of these facilities and similar outcomes among stroke survivors is recommended to measure improvements, if any, after this policy is fully enacted.

### Limitations

There were several limitations in the current study. The CHARLS study sample included community-based middle-aged and older adults, which excluded institutionalized individuals. Thus, survivors of stroke who were admitted to or readmitted to the hospital or were living in nursing homes were not included. Thus, these findings may only be generalized to community-dwelling survivors of stroke. The CHARLS asked the participants to self-report their social participation in the last month. Thus, recall bias may affect the results. Lastly, we did not include objectively measured performance-based indicators of physical capacity, such as grip strength, repeated chair stands, and a series of balance tests due to the severity of missing information across these indicators.

## 5. Conclusions

Our findings indicate that Chinese middle-aged and older adults post-stroke interact with friends and go to a community club to play table games most frequently. Social participation was not only associated with factors at the individual-level and community-level, but was also associated with physical disabilities and mental disorders. Difficulties with upper limb functioning were negatively affected when interacting with friends and difficulties with lower limb functioning were negatively affected when going to a community club to play table games. Thus, it is necessary to group rehabilitation professionals from interdisciplinary backgrounds to improve opportunities for social participation among stroke survivors. Although this study targeted the Chinese population, it can also inform the international community in that stroke rehabilitation should not only focus on physical and mental disabilities, but also on social isolation that can be caused by a lack of social participation. Moreover, cultural preference should be considered when assessing facilitators or barriers to participation in social activities among older stroke survivors.

## Figures and Tables

**Table 1:** Differences among key characteristics of stroke survivors between 2011 and 2015 (Mean or N (%)).

Sample Characteristics	2011 (N = 413)	2013 (N = 395)	2015 (N = 441)	$F/\chi^{2}$	*p*
**Demographic factors**					
Age	64.6/66.3	65.2/66.4	66.5/66.0	4.02	0.018
Male	216 (53.3)	207 (54.8)	234 (57.4)	0.06	0.971
Primary school and lower	291 (70.1)	273 (64.7)	292 (59.5)	1.87	0.392
Rural residency	282 (58.2)	273 (59.5)	318 (66.8)	1.65	0.438
Married and living with the spouse	317 (71.2)	314 (74.7)	336 (74.3)	1.46	0.483
**Health status**					
Poor self-rated health	278 (67.6)	230 (56.5)	260 (52.8)	8.88	0.012
No. of morbidities	2.4/2.5	2.5/2.6	2.7/2.7	4.42	0.012
Depressive symptoms	269 (68.7)	244 (64.3)	267 (63.3)	2.03	0.363
Needing assistance with ADLs	373 (88.5)	373 (93.8)	411 (94.2)	5.33	0.070
Needing assistance with IADLs	344 (79.8)	353 (89.1)	404 (93.5)	14.94	0.001
**Difficulty with**					
Running/jogging about 1 km	322 (79.5)	326 (81.3)	353 (73.0)	2.65	0.266
Walking 1 km	194 (47.1)	186 (45.8)	196 (40.0)	0.77	0.681
Walking 100 m	132 (35.2)	185 (44.7)	195 (38.2)	21.40	<0.001
Getting up from a chair	237 (57.2)	209 (51.2)	231 (45.8)	2.54	0.281
Climbing several flights of stairs without resting	303 (74.3)	267 (68.5)	282 (57.4)	8.83	0.012
Stooping, kneeling/crouching	255 (62.7)	238 (61.8)	251 (51.9)	2.18	0.337
Reaching/extending arms above shoulder level	141 (34.1)	103 (24.8)	147 (27.9)	7.41	0.025
Lifting/carrying weights over 5 kg	167 (41.8)	138 (34.5)	166 (37.0)	2.60	0.272
Picking up a small coin from a table	84 (21.1)	70 (18.1)	82 (17.6)	0.94	0.624
**Social participation**					
Mean of social activities participated	0.6/0.6	0.8/0.8	0.7/0.9	4.24	0.015
Types of social activity participated: 0	246 (62.1)	208 (52.4)	259 (55.0)	16.59	0.002
1	124 (26.5)	107 (28.3)	111 (21.6)		
>1	43 (10.3)	80 (19.3)	71 (23.4)		

Numerical variables presented two means: the first mean unweighted and the second weighted, categorical variables presented as n (%), with n being unweighted frequency and % being weighted as a column percentage.

**Table 2:** Social activities participated in among survivors of stroke (N (%)).

Type of Social Participations	2011 (N = 413)	2013 (N = 395)	2015 (N = 441)
Interacting with friends	103 (22.1)	121 (29.4)	123 (32.6)
Going to a community club to play table games	59 (12.6)	71 (16.9)	77 (22.7)
Providing help to individuals who do not live with you for free	17 (3.0)	42 (8.9)	38 (8.8)
Going to a sport, social, or other kind of club	33 (8.8)	38 (12.2)	18 (4.0)
Taking part in a community-related organization	8 (2.7)	11 (3.5)	8 (2.6)
Doing voluntary or charity work	2 (0.6)	5 (1.9)	4 (1.0)
Caring for a sick/disabled adult who does not live with you for free	3 (0.6)	8 (1.9)	9 (1.7)
Attending an educational or training course	3 (0.5)	3 (1.1)	1 (0.3)
Stock investment	1 (0.2)	1 (0.1)	3 (1.1)
Used the Internet	9 (2.9)	12 (3.2)	14 (1.1)
Others	4 (1.1)	4 (0.9)	6 (1.3)

Categorical variables presented as n (%), with n being unweighted frequency, and % being a weighted column percentage.

**Table 3:** The number of social activities participated in among stroke survivors with different health issues (Mean ± SD).

Stroke Survivors with the Following Health Issues	2011	2013	2015
Needing assistance with ADLs	0.64 ± 0.95	0.85 ± 1.16	0.73 ± 1.08
Needing assistance with IADLs	0.68 ± 0.98	0.88 ± 1.17	0.73 ± 1.09
Depressive symptoms	0.46 ± 0.85	0.57 ± 0.98	0.50 ± 0.87
Difficulty with running or jogging about 1 km	0.82 ± 1.09	1.07 ± 1.24	0.92 ± 1.18
Difficulty with climbing several flights of stairs without resting	1.01 ± 1.24	1.09 ± 1.39	0.99 ± 1.30
Difficulty with reaching or extending your arms above shoulder level	0.71 ± 1.04	0.98 ± 1.24	0.86 ± 1.18
Difficulty with lifting/carrying weights over 5 kg	0.78 ± 1.03	0.99 ± 1.26	0.91 ± 1.20

SD: Standard Deviation.

**Table 4:** Zero-inflated Poisson regression on the number of social activities that stroke survivors participated in.

Associated Factors with the Number of Social Activities Participated in	Coefficient (95% CI)	*Std. Err.*	*Z*	*p*
Needing assistance with ADLs	1.78 (0.75, 2.81)	0.524	3.39	0.001
Needing assistance with IADLs	0.98 (0.41, 1.56)	0.294	3.34	0.001
Depressive symptoms	−0.35 (−0.65, −0.06)	0.149	−2.36	0.019
Difficulty with running or jogging about 1 km	0.46 (0.14, 0.79)	0.165	2.80	0.005
Difficulty with climbing several flights of stairs without resting	0.29 (0.01, 0.58)	0.146	2.01	0.045
Difficulty with reaching or extending your arms above shoulder level	0.32 (0.08, 0.57)	0.125	2.60	0.010
Difficulty with lifting/carrying weights over 5 kg	0.29 (0.07, 0.52)	0.113	2.59	0.010
***Inflate***				
Primary school or lower	1.01 (0.16, 1.87)	0.434	2.33	0.020

Coef.: Coefficient. CI: Confidential Interval.

**Table 5:** Multi-level logistic regression on the probability of types of social activities participated in.

Multi-Level Factors Associated with the Probability of Social Participation	Participating in Any Social Activity versus None		Interacting with Friends		Going to a Community Club to Play Table Games	
	OR (95% CI)	*p*	OR (95% CI)	*p*	OR (95% CI)	*p*
**Socio-demographic factors**						
Age					0.97 (0.95, 0.99)	0.022
Primary school or lower					0.53 (0.33, 0.86)	0.009
Male			0.62 (0.46, 0.83)	0.002		
Married and living with the spouse			0.73 (0.51, 1.05)	0.087		
**Health-related factors**						
Needing assistance with ADLs	5.53 (1.84, 16.56)	0.002	4.57 (1.32, 15.89)	0.017		
Needing assistance with IADLs	2.95 (1.52, 5.72)	0.001	2.12 (1.03, 4.39)	0.042		
No. of morbidities						
Depressive symptoms	0.50 (0.37, 0.67)	<0.001	0.72 (0.53, 0.99)	0.042	0.36 (0.23, 0.57)	<0.001
**Difficulty with**						
Walking 1 km	0.63 (0.46, 0.86)	0.004	0.58 (0.41, 0.82)	0.002		
Walking 100 m			1.49 (1.05, 2.08)	0.023		
Stooping/kneeling/crouching			0.69 (0.50, 0.96)	0.027		
Reaching/extending arms above shoulder level					0.30 (0.17, 0.55)	<0.001
Lifting/carrying weights over 5 kg	0.50 (0.35, 0.69)	<0.001	0.58 (0.40, 0.84)	0.004	0.54 (0.32, 0.91)	<0.001
Picking up a small coin from a table					0.50 (0.28, 0.81)	0.006
**Community-based**						
Allocating the outdoor exercise facility in the community/village					1.51 (1.17, 1.96)	<0.001
AIC	1482.15		1352.61		921.41	
BIC	1523.12		1408.94		962.38	
ICC	0.17		0.14		0.41	
